# Emerging strategies in colorectal cancer immunotherapy: enhancing efficacy and survival

**DOI:** 10.3389/fimmu.2025.1616414

**Published:** 2025-10-02

**Authors:** Yu Zhang, Haixia Guan, Xixuan Feng, Mengyan Liu, Jinhuan Shao, Mengchi Liu, Jialei He, Yahui Jin, Jinglin Zhu, Chunli Zheng

**Affiliations:** Yan’an Medical College, Yan’an University, Yan’an, Shaanxi, China

**Keywords:** colorectal cancer immunotherapy, immune checkpoint inhibitors, cancer vaccines, adoptive cell therapy, matrix-depletion therapy

## Abstract

Colorectal cancer (CRC) is a prevalent malignancy of the digestive system, with metastatic CRC (mCRC) exhibiting persistently poor overall survival rates. Consequently, there is an urgent need to develop more effective and safer therapeutic strategies. In recent years, immunotherapy has emerged as a groundbreaking approach in CRC treatment. This review highlights the advancements in immune checkpoint Inhibitors (ICIs), cancer vaccines, oncolytic virotherapy, adoptive cell therapy(ACT), and matrix-depletion therapy. Additionally, we explore potential combinatorial immunotherapy strategies for CRC, emphasizing their clinical applications and addressing the challenges associated with CRC immunotherapy. By proposing strategies to overcome these limitations, this review aims to provide novel insights into the evolving landscape of CRC immunotherapy.

## 1 Introduction

CRC ranks as the third most common cancer globally and is the second leading cause of cancer-related mortality. Despite significant advances in diagnostic methods and treatment options, the prognosis for patients with mCRC continues to be poor, particularly for those diagnosed with late-stage disease. While early-stage CRC (stage I) exhibits a 5-year survival rate of 91%, it dramatically declines to approximately 14% for patients with metastatic involvement (1), emphasizing the urgent need for more effective therapeutic strategies. Traditional treatment modalities such as surgery, radiotherapy, and chemotherapy have demonstrated limited efficacy in advanced CRC, primarily due to the inherent tumor heterogeneity, the emergence of resistance mechanisms, and the lack of durable responses (2–5). More recently, immunotherapy has emerged as a promising fourth pillar in cancer treatment, complementing conventional therapies and offering substantial benefits in specific patient populations (6–9). Among these, ICIs have revolutionized the treatment landscape for solid tumors, including CRC (10). Additionally, personalized therapeutic strategies such as cancer vaccines and oncolytic virotherapy are under active investigation (11, 12). Yet, the application of these approaches is associated with a range of benefits and drawback(**Table 1**).

**Table 1:** Comparative analysis and rationale for included immunotherapy groups in colorectal cancer.

Immunotherapy strategies	Advantages	Disadvantages/limitations	Current status in CRC	Rationale for inclusion in review
PD-1/PD-L1 Inhibitors	Durable responses in MSI-H/dMMR subset; Foundation of many combination strategies; Multiple approved agents (Nivo, Pembro).	Limited efficacy in MSS/pMMR CRC; Primary/acquired resistance common; Immune-related adverse events (irAEs).	Established: FDA-approved for MSI-H/dMMR mCRC. Active research in combinations & MSS setting.	Cornerstone of CRC immunotherapy; essential baseline for comparison; significant clinical impact in defined subset.
CTLA-4 Inhibitors	Potential to overcome resistance to PD-1/PD-L1; CTLA-4 crucial for priming T-cell response; Wide range of use.	Significantly higher toxicity (esp. colitis); Limited single-agent activity; Efficacy mostly in combo with ICIs.	Established Combo (CTLA-4):Approved in MSI-H mCRC; The optimal combination regimen and patient selection criteria are unclear	Critical for understanding combinatorial strategies; High toxicity profile analysis.
LAG-3 Inhibitors	LAG-3 emerging as potent combo target; Good tolerability; Fewer adverse events.	Clinical data are scarce; Lack of reliable predictive markers; Efficacy mostly in combo with PD-1 inhibitors.	Emerging (LAG-3):Phase 3 ongoing; The efficacy needs to be further verified.	An emerging immune checkpoint inhibitor; The combination with PD-1 inhibitors is of interest.
Neoantigen Vaccines	Highly personalized targeting; Potential synergy with ICIs; Induce durable T-cell responses.	Complex/expensive manufacturing; Requires tumor sequencing; Limited patient accessibility.	Early Experimental: Predominantly Phase 1/2. No approvals.	Forefront of personalized immunotherapy; Potential for MSS tumors; Critical for future directions.
Other Immunotherapy	Novel mechanisms overcoming ICIs resistance; ACT offers personalized approach; Targets tumor microenvironment.	High complexity & cost; Early-stage data predominates; Logistical challenges.	Experimental: Mostly Phase 1/2; No widespread approvals.	Represents non-ICI approaches; Crucial for understanding broader landscape; High potential but high hurdles.

CRC has a unique tumor microenvironment (TME) compared to other cancer types, primarily due to microsatellite instability (MSI), which is often a phenotypic consequence of mismatch repair-deficient (dMMR) (13). In dMMR/MSI-H CRC, the TME is enriched with immune cells, particularly CD8^+^ T cells, driven by high tumor mutation burden (TMB) and neoantigen production (14, 15). These factors create an inflammatory TME, supporting the efficacy of ICIs like programmed death-1 (PD-1)/programmed death-ligand 1 (PD-L1) blockers. PD-L1 expression is also elevated in dMMR/MSI-H CRC, further enhancing ICIs effectiveness. However, immune suppressive elements, including Tregs, MDSCs, and factors like TGF-β and IL-10, can limit ICIs responses, causing resistance (13). Conversely, mismatch repair-proficient (pMMR)/MSS CRC shows sparse immune infiltration and low PD-L1 expression, leading to intrinsic ICIs resistance. Its TME may harbor more immunosuppressive factors, reducing immunotherapy efficacy (16). This contrast underscores the need for strategies to counteract the immunosuppressive TME, aiming to extend immunotherapy benefits to 85% of MSS CRC patients. Transforming “cold” into “hot” tumors mainly involves combining checkpoint inhibitors with treatments like radiotherapy, chemotherapy, and anti-angiogenic drugs. Other strategies include boosting tumor cell immunogenicity, enhancing antigen presentation, recruiting/activating immune cells, and reprogramming the TME. While early clinical trials show promise, challenges such as TME complexity and patient heterogeneity persist. Further research is crucial to optimize these approaches and enhance immunotherapy efficacy in a broader patient population.

**Figure 1:**
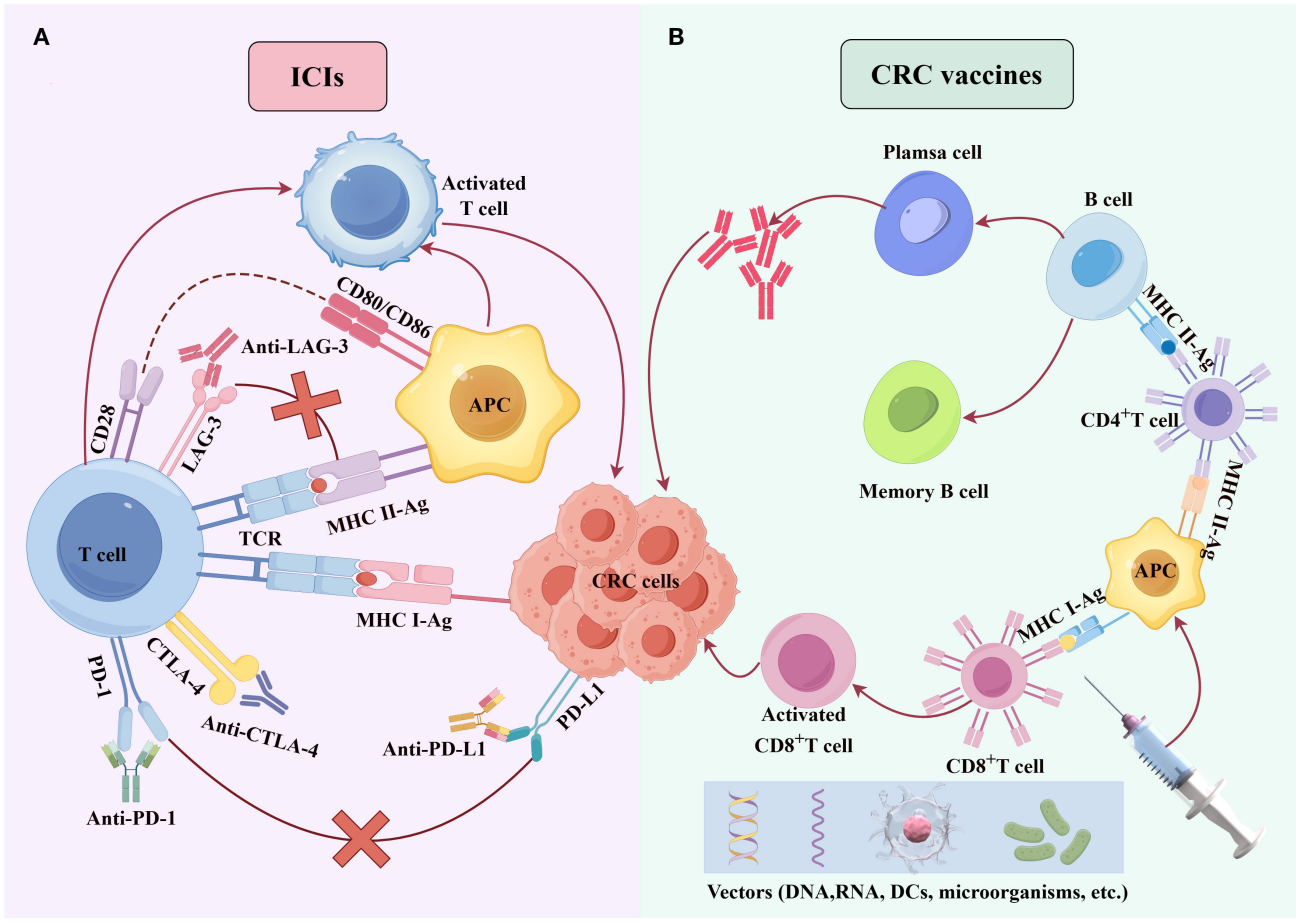
Molecular mechanisms of ICIs and cancer vaccines. **(A)** PD-1/PD-L1/LAG-3 inhibitors block the binding of PD-1/PD-L1/LAG-3 binding, counteracting CRC cell-mediated immune suppression of T cells and enhancing T-cell-mediated cytotoxicity against CRC cells. **(B)** Tumor antigens, delivered via diverse vectors (e.g., DNA, RNA, DCs, or microbes), are processed by dendritic cells, presented to T cells, and activate antigen-specific cytotoxic T cells to eliminate CRC cells.

**Figure 2:**
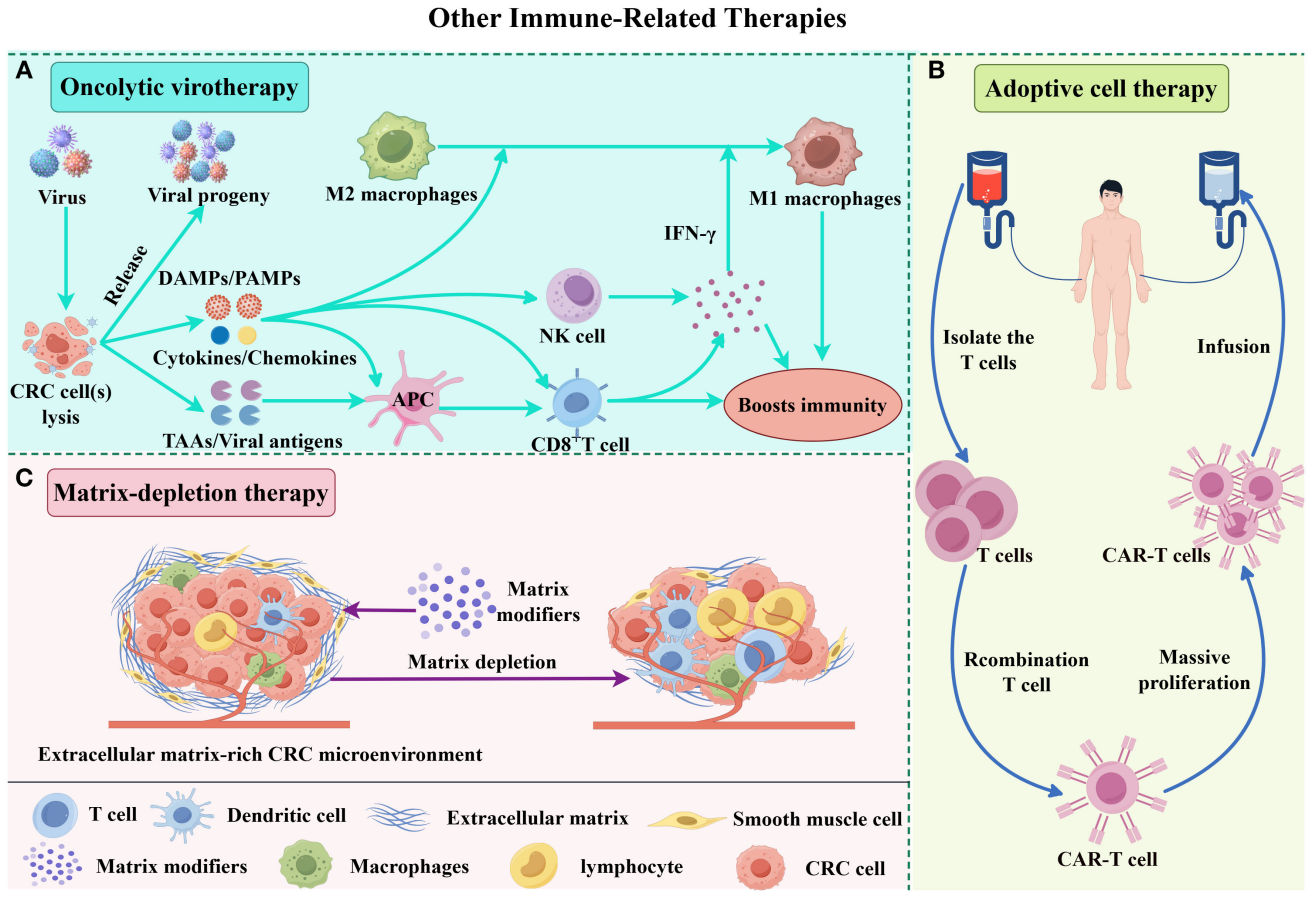
Molecular mechanisms of other immune-related combination therapies. **(A)** Upon infection by an oncolytic virus, CRC cells mount an antiviral response, secreting antiviral cytokines-particularly interferons (IFNs). These cytokines promote the maturation of antigen-presenting cells (APCs), such as dendritic cells (DCs), and stimulate CD8^+^ T cells and natural killer (NK) cells. As infected tumor cells lyse, they release viral progeny, damage-associated molecular patterns (DAMPs) like host cell proteins, pathogen-associated molecular patterns (PAMPs) from viral particles, and tumor-associated antigens (TAAs), including neoantigens. The viral progeny then go on to infect additional tumor cells. DAMPs and PAMPs activate the immune system via receptors like Toll-like receptors (TLRs). Meanwhile, APCs capture TAAs and neoantigens, triggering antigen- and virus-specific CD8^+^ T cell responses. This cascade creates an immune-stimulatory environment that drives tumor-supportive M2 macrophages to shift toward a pro-inflammatory M1 phenotype. **(B)** ACT involves isolating autologous T/NK cells, ex vivo expansion and/or genetic engineering to enhance tumor specificity, followed by reinfusion. These activated lymphocytes selectively recognize and eliminate CRC cells. **(C)** Matrix modulators deplete extracellular matrix components, disrupting the tumor microenvironment to enhance immune cell infiltration and drug penetration, thereby potentiating therapeutic efficacy.

In this review, we explore the molecular mechanisms underlying immune evasion in CRC, focusing on immune checkpoint inhibitors, cancer vaccines, oncolytic virotherapy, ACT, and matrix-depletion therapy (**Figures 1**, **2**). We also discuss current challenges in CRC immunotherapy, including resistance mechanisms, limited clinical efficacy in MSS tumors, and the need for personalized treatment approaches. The review aims to provide a comprehensive overview of the current state of immunotherapy in CRC and highlight emerging strategies aimed at overcoming these barriers, ultimately improving patient outcomes.

## 2 ICIs

ICIs represent a paradigm shift in cancer immunotherapy, functioning by reversing immune suppression mediated by checkpoint molecules and reactivating T-cell-mediated antitumor responses (17, 18). The PD-1/PD-L1 axis and cytotoxic T-lymphocyte-associated antigen-4 (CTLA-4) have emerged as key targets in the treatment of various cancers, including CRC, demonstrating significant clinical efficacy (19, 20). More recently, lymphocyte activation gene 3 (LAG-3) has emerged as a next-generation immune checkpoint, offering promising potential for advancing cancer therapy (21).

PD-1, CTLA-4, and LAG-3 serve as negative regulators of T-cell activation and migration, playing pivotal roles in immune checkpoint blockade (10, 22). As such, inhibitors targeting these checkpoints have been pivotal in immune checkpoint blockade strategies, significantly enhancing immune-mediated tumor clearance and inducing durable responses across a variety of solid tumors and hematologic malignancies (23, 24). Notably, combination therapies involving LAG-3 inhibitors alongside PD-1/PD-L1 blockade have shown synergistic effects, further enhancing therapeutic outcomes in preclinical and clinical settings (25–28). Despite these promising developments, the clinical application of these inhibitors is not without challenges. Although PD−1/PD−L1 or CTLA−4 blockade significantly prolongs overall survival in patients with PD−L1–positive advanced non–small cell lung cancer (NSCLC), unresectable stage III–IV melanoma, and CRC—with the greatest benefit observed in dMMR/MSI−H CRC—these therapies can also induce immune−related adverse events (irAEs) in a subset of patients, potentially limiting their broader applicability (29–32). Furthermore, the development of resistance to these therapies remains a significant hurdle, with some patients exhibiting primary or acquired resistance (33, 34), underscoring the need for more personalized treatment strategies that consider individual tumor profiles and immune landscape variations. This section aims to provide a detailed overview of the mechanisms of action, clinical applications, and current limitations of PD-1/PD-L1, CTLA-4, and LAG-3 inhibitors, as well as the strategies being explored to overcome these barriers.

### 2.1 Anti-PD-1 and anti-PD-L1 inhibitors

PD−1 is an inhibitory receptor of the B7−CD28 family, expressed on activated T cells, B cells, and a subset of NK cells. Upon engagement by PD−L1, tyrosine residues within PD−1’s ITSM and ITIM motifs are phosphorylated, creating docking sites for SHP2. In its basal state, SHP2’s N−SH2 domain occludes its PTP catalytic pocket; ligand−induced phosphotyrosine binding displaces N−SH2, unleashing phosphatase activity (35). Activated SHP2 preferentially dephosphorylates the co−stimulatory receptor CD28—rather than TCR components such as CD3ζ or ZAP70—thereby attenuating downstream PI3K/Akt signaling, IL−2 production, and T−cell proliferation (36). In myeloid cells, PD−1–SHP2 signaling also impairs phosphorylation of transcription factors IRF8 and HOXA10, inhibiting monocyte differentiation and antigen presentation; intriguingly, SHP2 appears dispensable for PD−1–mediated T−cell exhaustion in chronic viral infection models, underscoring context−dependent pathway regulation (37, 38). Furthermore, numerous studies have established a correlation between PD-L1 upregulation and poor prognosis in CRC (39–41). PD-1/PD-L1 inhibitors counteract this immune suppression by blocking the PD-1/PD-L1 pathway, thereby enhancing T-cell-mediated antitumor immunity. To date, the U.S. Food and Drug Administration (FDA) has approved several PD-1 (**Table 2**) /PD-L1(**Table 3**) inhibitors, including pembrolizumab, nivolumab, cemiplimab, and avelumab, for the treatment of various malignancies (42).

**Table 2:** Clinical trials based on PD-1 inhibitors in the treatment of colorectal cancer.

Angent	ClinicalTrials. Gov Identifier	Combinatorial agent(s)	Phase	Primary endpoint	Status
Nivolumab	NCT04943900	BMS-986416	Phase 1	TEAEs,MTD	Active
Nivolumab	NCT04895709	Docetaxel;BMS-986340; BMS-936558-01	Phase 1,2	TEAEs	Recruiting
Nivolumab	NCT02327078	Epacadostat	Phase 1,2	DLTs,TEAEs,ORR,PFS	Completed
Nivolumab	NCT03647839	BNC105;BBI-608	Phase 2	OR	Completed
Nivolumab	NCT03785210	Tadalafil;Vancomycin	Phase 2	BOR	Completed
Nivolumab	NCT02860546	TAS-102	Phase 2	irORR	Completed
Nivolumab	NCT05061017	Low-dose Cyclophosphamide	Phase 2	ORR	Completed
Nivolumab	NCT03414983	Oxaliplatin;Leucovorin	Phase 2,3	PFS	Completed
Nivolumab	NCT04008030	Ipilimumab	Phase 3	PFS	Active
Pembrolizumab	NCT03332498	Ibrutinib	Phase 1,2	RP2D,DCR	Completed
Pembrolizumab	NCT03168139	Olaptesed (NOX-A12)	Phase 1,2	Safety,DCR,PFS,OS	Completed
Pembrolizumab	NCT02437136	Entinostat	Phase 1,2	Safety,OR,ORR	Completed
Pembrolizumab	NCT03631407	Vicriviroc	Phase 2	ORR,DLTs	Completed
Pembrolizumab	NCT03797326	Lenvatinib(E7080/MK-7902)	Phase 2	ORR,TEAEs	Completed
Pembrolizumab	NCT02460198		Phase 2	ORR	Completed
Pembrolizumab	NCT04776148	Lenvatinib(E7080/MK-7902)	Phase 3	OS	Completed
Pembrolizumab	NCT02563002	mFOLFOX6;FOLFIRI	Phase 3	PFS,OS	Completed
Pembrolizumab	NCT05239741	Oxaliplatin;Leucovorin	Phase 3	PFS	Recruiting
Pembrolizumab	NCT06550453	Bevacizumab;CapeOX	Phase 4	pCR	Recruiting
Spartalizumab	NCT02947165	NIS793	Phase 1	DLTs,TEAEs	Completed
Spartalizumab	NCT02890069	LCL161;Everolimus;Panobinostat	Phase 1	DLTs,TEAEs	Completed
Tisleizumab-BGB-A317	NCT06262581		Phase 2	pCR	Recruiting
Cemiplimab	NCT06205836	Fianlimab	Phase 2	CRR	Recruiting

TEAEs, Treatment-Emergent Adverse Events; MTD, Maximum Tolerated Dose; DLTs, Dose-Limiting Toxicities; ORR, Objective Response Rate; PFS, Progression-Free Survival; OR, Overall Response; BOR, Best Overall Response; irORR, Immune-related Objective Response Rate; RP2D, Recommended Phase 2 Dose; DCR, Disease Control Rate; OS, Overall Survival.

**Table 3:** Clinical trials based on PD-L1 inhibitors in the treatment of colorectal cancer.

Angent	ClinicalTrials.Gov Identifier	Combinatorial agent(s)	Phase	Primary endpoint	Status
Durvalumab	NCT02754856	Tremelimumab	Phase 1	Post-operative Toxicity,Safety,Feasibility	Completed
Durvalumab	NCT03005002	Tremelimumab	Phase 1	RECIST,TEAEs	Completed
Atezolizumab	NCT03841110	FT500;Nivolumab;Pembrolizumab; Cyclophosphamide;Fludarabine;IL-2	Phase 1	DLTs,MTD,MAD	Completed
Atezolizumab	NCT04713891	KF-0210	Phase 1	DLTs,MTD,Safety	Completed
Atezolizumab	NCT02876224	Cobimetinib;Bevacizumab	Phase 1	TEAEs	Completed
Atezolizumab	NCT03256344	Talimogene Laherparepvec	Phase 1	DLTs,ORR,BOR,DOR	Completed
Atezolizumab	NCT02873195	Bevacizumab;Capecitabine	Phase 2	PFS,OS,ORR	Completed
Atezolizumab	NCT03721653	FOLFOXIRI;Bevacizumab	Phase 2	PFS	Completed
Atezolizumab	NCT02291289	FOLFOX;Cetuximab	Phase 2	PFS	Completed
Atezolizumab	NCT02788279	Cobimetinib;Regorafenib;	Phase 3	OS	Completed
Avelumab	NCT01772004		Phase 1	DLTs,BOR	Completed
Avelumab	NCT03152565	Autologous Dendritic Cell Vaccine	Phase 1,2	PFS,Does	Completed
Avelumab	NCT03258398	eFT508	Phase 2	DLTs	Completed
Avelumab	NCT03854799	Capecitabine	Phase 2	pCR	Completed
Avelumab	NCT03174405	Cetuximab; FOLFOX	Phase 2	PFS	Completed
Avelumab	NCT04513951	Cetuximab;mFOLFOXIRI	Phase 2	PFS	Completed
Avelumab	NCT04561336	Cetuximab	Phase 2	OS	Completed

MAD, Maximum Administered Dose; DOR Duration of Response.

The KEYNOTE-177 trial, a landmark international Phase III study, has drawn significant attention for its evaluation of pembrolizumab versus chemotherapy in patients with dMMR/MSI-H mCRC (32). The primary endpoints were OS and PFS. In the final analysis, the median PFS was 16.5 months for pembrolizumab monotherapy compared to 8.2 months for chemotherapy, although no statistically significant difference in OS was observed between the two groups (32). The favorable safety profile of pembrolizumab, as demonstrated in prior trials, further supports its clinical utility (43). These findings underscore the superiority of pembrolizumab as a first-line treatment for dMMR/MSI-H mCRC, offering prolonged PFS and reduced adverse events compared to conventional chemotherapy. Consequently, the FDA approved pembrolizumab for this indication, marking a significant milestone in CRC immunotherapy.

The Phase II CheckMate 142 trial evaluated the efficacy of nivolumab combined with relatlimab, a LAG-3 inhibitor, in patients with dMMR/MSI-H mCRC (44). After a median follow-up of 47.4 months, the ORR, DCR, median PFS, and 2-year PFS rate were 50%, 70%, 27.5 months, and 51%, respectively (44). Compared to pembrolizumab or nivolumab monotherapy, the combination of nivolumab and relatlimab yielded a higher ORR and a longer investigator-assessed PFS, highlighting the durable clinical benefits of this dual checkpoint blockade (43, 45). These results suggest that combining nivolumab with relatlimab represents a promising therapeutic strategy for dMMR/MSI-H mCRC.

Notably, two neoadjuvant trials illustrate the potential for combining immune checkpoint blockade with other modalities. In the randomized phase II PICC trial (NCT03926338), 34 patients with locally advanced dMMR/MSI-H CRC received neoadjuvant toripalimab either alone or alongside the COX-2 inhibitor celecoxib; the combination arm achieved an impressive pCR rate of 88% versus 65% with toripalimab alone, and no relapses were observed at a 12-month follow-up, with only 3% of patients experiencing grade ≥ 3 immune-related adverse events (46). In another ongoing phase II study (NCT04715633), 52 participants with dMMR/MSI-H CRC are being treated preoperatively with camrelizumab plus oral apatinib; interim analyses report an ORR of 42% and a favorable safety profile, with mature pCR and long−term outcomes eagerly awaited. Another ongoing phase II trial NCT06205836 is investigating the safety and efficacy of cemiplimab, both as a monotherapy and in combination with fianlimab, in patients aged 70 years and older with dMMR/MSI-H mCRC. The primary endpoint is the complete response rate (CRR), and the results are highly anticipated.

### 2.2 CTLA-4 inhibitors

Structurally, CTLA-4 has a high homology with CD28, but it has a greater affinity for B7 homologs than CD28: CTLA-4 has a monomeric affinity of about 0.2 μM for CD80 and a monomeric affinity of about 2 μM for CD86 (47). During transendocytosis, CD80 continues to bind to CTLA-4 and is endocytosed with CTLA-4 into advanced endosomes and lysosomes, inducing ubiquitination of lysine residues of CTLA-4, which ultimately leads to the degradation of CTLA-4, thereby reducing the amount of CTLA-4 on the cell surface, inhibiting the recycling of CTLA-4 and weakening its negative regulatory effect on the immune response (47). However, CD86 will rapidly dissociate and degrade after being captured by CTLA-4 and transendocytosis, while CTLA-4 will not be modified and can be recycled to the cell surface for further ligand capture, thus maintaining the negative regulatory function of CTLA-4 on the immune response to a certain extent (47). PD-L1 and CD80 can undergo cis-interaction within the cell, i.e., on the same cell surface, PD-L1 and CD80 can form heterodimers. This cis-interaction is able to inhibit the binding of PD-L1 to PD-1 and the interaction of CD80 with CTLA-4, thereby enhancing the immune response to some extent (48). In summary, CTLA-4 inhibitors may be used to treat CRC with a combination of drugs that modulate CD80 and CD86 expression, or in combination with PD-L1 inhibitors, may achieve better therapeutic outcomes (49). At the same time, according to the individual differences of patients (such as the expression levels of CD80, CD86 and PD-L1 on the surface of tumor cells), personalized combination treatment plans are formulated to improve the pertinence and effectiveness of treatment.

Leveraging the molecular choreography of CTLA-4, CD28, and PD-L1 offers a blueprint for precision therapy in CRC. Tumors with dominant CD80 signaling—readily stripped by CTLA-4 and routed to lysosomal oblivion—are primed for CTLA-4 blockade, whereas CD86-centric lesions, whose ligand dissociates and spares CTLA-4 recycling, may demand dual checkpoint inhibition to extinguish residual negative feedback. Concomitant quantification of PD-L1-CD80 cis-heterodimers on tumor cells stratifies patients further: where this inhibitory pair is abundant, adding a PD-L1 antagonist liberates both CD80 for CD28 co-stimulation and PD-L1 from PD-1 restraint, amplifying anti-tumor immunity. Thus, an integrative map of surface CD80, CD86, and PD-L1 densities—together with germline CTLA-4 variants that tune ligand avidity—can guide calibrated sequencing or combination of CTLA-4 and PD-L1 inhibitors, converting mechanistic insight into bespoke regimens that maximize efficacy while minimizing collateral toxicity. These clinical studies will provide key insights into the treatment of CRC (**Table 4**).

**Table 4:** Clinical trials based on CTLA-4 inhibitors in the treatment of colorectal cancer.

Angent	ClinicalTrials.Gov Identifier	Combinatorial agent(s)	Phase	Primary endpoint	Status
Ipilimumab	NCT05909995	INCB 99280	Phase 1	DLTs,TEAEs	Terminated
Ipilimumab	NCT04721301	Maraviroc;Nivolumab	Phase 1	TEAEs	Completed
Ipilimumab	NCT03507699	Radiosurgery;CMP-001; Radiosurgery;Nivolumab	Phase 1	DLTs	Completed
Ipilimumab	NCT03241173	INCAGN01949; Nivolumab	Phase 1,2	TEAEs,ORR	Completed
Ipilimumab	NCT04730544	Nivolumab	Phase 2	TEAEs,PFS	Recruiting
Ipilimumab	NCT03755739	Chemotherapy	Phase 2,3	OS,pCR	Recruiting
Ipilimumab	NCT04157985	Nivolumab;Pembrolizumab;Atezolizumab	Phase 3	Time to next treatment,PFS	Recruiting
Tremelimumab	NCT02586987	Selumetinib;MEDI4736	Phase 1	Safety,Tolerability	Completed
Tremelimumab	NCT01975831	MEDI4736	Phase 1	Safety,MTD	Completed
Tremelimumab	NCT03539822	Durvalumab;Cabozantinib	Phase 1,2	MTD,ORR	Recruiting
Tremelimumab	NCT03202758	Durvalumab;FOLFOX	Phase 1,2	Safety	Completed
Tremelimumab	NCT03122509	Durvalumab;Radiotherapy;Ablation	Phase 2	ORR	Completed
Tremelimumab	NCT03007407	Durvalumab	Phase 2	ORR	Completed
Relatlimab	NCT05176483	Ipilimumab;Nivolumab	Phase 1	TEAEs,ORR,PFS,OS	Recruiting
Relatlimab	NCT03642067	Nivolumab	Phase 2	ORR	Completed
Relatlimab	NCT03867799	Nivolumab	Phase 2	DCR	Active
Relatlimab	NCT05328908	Nivolumab;Regorafenib	Phase 3	OS	Active
Favezelimab	NCT05064059	Pembrolizumab;Regorafenib;TAS-102	Phase 3	OS	Completed
Favezelimab	NCT05600309	Pembrolizumab;Regorafenib;TAS-102	Phase 3	OS	Completed

The TME harbors a significant population of regulatory T cells (Tregs), which express elevated levels of CTLA-4 and contribute to immunosuppression. In this context, CTLA-4 inhibitors, including ipilimumab and tremelimumab, have been developed and widely utilized in clinical practice. These agents exert their antitumor effects by alleviating T-cell suppression and depleting Tregs (50, 51). Building on this, Guo et al. (52) conducted a preclinical study on 2MW4691, a bispecific antibody designed to balance Treg depletion and T-cell activation. The experimental results suggest that 2MW4691 holds promise as a candidate for cancer therapy, warranting further evaluation of its tolerability in clinical trials.

In the CheckMate 142 trial, the combination of nivolumab with low-dose ipilimumab demonstrated a high response rate and reliable safety in 119 patients with dMMR/MSI-H CRC (53). After a median follow-up of 4 years, the ORR for the combination therapy was 65%, with a remarkable 48-month OS rate of 71%, underscoring the durable efficacy of this regimen (54). Another large-scale Phase III trial, CheckMate 8HW, enrolled 707 patients to evaluate the PFS of nivolumab plus ipilimumab versus nivolumab monotherapy in dMMR/MSI-H CRC. Across all treatment lines, the combination of nivolumab and ipilimumab outperformed nivolumab alone, offering a novel therapeutic option for patients with mCRC (55).

Tremelimumab, another well-characterized CTLA-4 inhibitor, differs from ipilimumab as an IgG2 monoclonal antibody. It has demonstrated promising therapeutic efficacy across various malignancies, including lung cancer (56), biliary tract cancer (57), and urothelial carcinoma (58). In a Phase Ib/II trial, the combination of tremelimumab and durvalumab with chemotherapy exhibited robust clinical activity in RAS-mutated mCRC (59). No safety concerns were observed in the Phase Ib portion, and the subsequent Phase II trial primarily assessed PFS, reporting a 3-month PFS rate of 90.7% and a secondary ORR of 64.5% (59).

### 2.3 LAG-3 inhibitors

LAG-3, a next-generation immune checkpoint, is highly expressed on exhausted T cells and has emerged as a promising therapeutic target. Initially identified for its ability to bind MHC class II molecules and inhibit T-cell activation (60). However, even in the absence of MHC II, LAG-3 binds to the TCR-CD3 complex and traces immune synapses, subsequently disrupting the binding of the tyrosine kinase p56lck (Lck) to CD4 and CD8 co-receptors, thereby limiting TCR signaling and downstream T cell activation (61). This effect is mediated by the EP motif, suggesting that TCR or CD3 is the primary cis-ligand for LAG-3 (61). LAG-3 regulates T-cell activation through steric modulation of antigen presentation: LAG-3 dimers compress the lateral spacing of MHC-II molecules on dendritic cells, occluding the CD4 co-receptor docking site and hindering TCR triggering (62). Epitope mapping of clinical-stage LAG-3 antagonists has revealed that blockade can be achieved without directly targeting the MHC-binding interface, suggesting alternative mechanisms to disrupt this inhibitory axis (62). These mechanistic insights not only deepen our understanding of checkpoint biology but also inform the design of next−generation immunotherapeutics. Furthermore, dual deficiency of PD-1 and LAG-3 in CD8^+^ T cells enhances tumor clearance, accompanied by increased interferon-γ (IFN-γ) release and upregulation of interferon-responsive genes (63).

The importance of alternative ligands such as galectin-3 (Gal-3), fibrinogen-like protein 1 (FGL1), LSECtin, and α-syn PFF needs to be further confirmed (64). Despite ongoing debates regarding LAG-3 signaling pathways and ligand interactions, the clinical synergy between LAG-3 inhibitors and other immune checkpoint blockers has been well-documented (65) (**Table 5**). Relatlimab (a LAG-3 monoclonal antibody) and nivolumab has received FDA approval for the treatment of unresectable or metastatic melanoma, marking a significant milestone in cancer immunotherapy (66).

**Table 5:** Clinical trials based on LAG-3 inhibitors in the treatment of colorectal cancer.

Angent	ClinicalTrials.Gov Identifier	Combinatorial agent(s)	Phase	Primary endpoint	Status
Relatlimab	NCT05176483	Ipilimumab;Nivolumab	Phase 1	TEAEs,ORR,PFS,OS	Recruiting
Relatlimab	NCT03642067	Nivolumab	Phase 2	ORR	Completed
Relatlimab	NCT03867799	Nivolumab	Phase 2	DCR	Active
Relatlimab	NCT05328908	Nivolumab	Phase 3	OS	Active
Favezelimab	NCT05064059	Pembrolizumab;Regorafenib;TAS-102	Phase 3	OS	Completed
Favezelimab	NCT05600309	Pembrolizumab;Regorafenib;TAS-102	Phase 3	OS	Completed
TSR-033	NCT02817633	TSR-022;TSR-042	Phase1	DLTs,TEAEs,ORR	Recruiting
TSR-033	NCT03250832	Dostarlimab	Phase 1	DLTs	Completed

In the context of CRC, the combination of nivolumab and relatlimab has demonstrated clinical benefits in patients with dMMR/MSI-H CRC, as evidenced by the CheckMate 142 trial (44). A Phase III trial evaluating favezelimab (a LAG-3 inhibitor) plus pembrolizumab in CRC reported no treatment-related deaths and notable antitumor activity in PD-L1-positive patients (67). Additionally, the global KEYFORM-007 trial is investigating the safety and efficacy of the co-formulated favezelimab/pembrolizumab (MK-4280A) in PD-L1-positive CRC patients, with an enrollment of 505 participants. This trial also compares the combination to standard-of-care treatments such as TAS-102 (trifluridine and tipiracil) and regorafenib, with results eagerly anticipated. Tebotelimab, a bispecific antibody targeting both PD-1 and LAG-3, has shown encouraging therapeutic responses in solid tumors and hematologic malignancies, with an ORR of 19% and a favorable safety profile (68). These findings highlight the potential of dual checkpoint blockade in overcoming immune resistance and enhancing antitumor immunity.

Currently, ICIs show more efficacy in dMMR/MSI-H CRC patients. However, the heterogeneity of CRC can restrict ICIs’ effectiveness. Moreover, ICIs’ use often leads to drug resistance and immune-related side effects. Thus, a comprehensive patient evaluation before treatment is necessary. Since ICIs have limited efficacy in pMMR/MSS CRC patients, they usually need to be used with other therapies.

## 3 Exploration of immunotherapy in pMMR/MSS CRC

Compared to dMMR/MSI-H CRC, pMMR/MSS CRC is characterized by a “cold” immune microenvironment, with limited immune cell infiltration and low TMB. This results in minimal benefits from monotherapy with immune checkpoint inhibitors, necessitating the development of combination strategies to overcome immune resistance (69). Below, we discuss several promising approaches to enhance immunotherapy efficacy in pMMR/MSS CRC.

### 3.1 ICIs plus chemotherapy

Chemotherapeutic agents, such as oxaliplatin and 5-fluorouracil, can induce immunogenic cell death, releasing tumor antigens and neoantigens that activate dendritic cells and enhance antigen presentation (70, 71). Additionally, chemotherapy modulates the immunosuppressive TME by reducing the population of Tregs and myeloid-derived suppressor cells (MDSCs) while increasing CD8^+^ T-cell infiltration, thereby converting “cold” tumors into “hot” tumors (72, 73). Furthermore, chemotherapy may upregulate PD-L1 expression on tumor cells, enhancing the targeting efficacy of ICIs (74, 75). This synergistic interplay between chemotherapy and ICIs creates a dual mechanism of “immune priming and immune maintenance,” offering a promising strategy for pMMR/MSS CRC.

The KEYNOTE-651 study evaluated the long-term safety and efficacy of pembrolizumab combined with mFOLFOX7/FOLFIRI in pMMR/MSS CRC patients, demonstrating favorable outcomes in Cohorts A, C, and E (76). In Cohorts B and D, the benefit of ORR appeared more pronounced in the KRAS wild-type subgroup, warranting further investigation (77). Another multicenter Phase II trial, GOIM 2802, showed that bevacizumab combined with the XELOX-2 regimen administered biweekly was effective and well-tolerated in mCRC patients, with an ORR comparable to that of the bevacizumab plus FOLFOX-4 group (78). The CAMILLA trial (NCT03539822), a Phase I/II clinical study, established the safety of cabozantinib plus durvalumab combined with chemotherapy in pMMR/MSS mCRC patients during its Phase I component (79). The Phase II portion, which included a CRC cohort, reported a DCR of 86.2%, highlighting the antitumor activity and manageable toxicity of this combination (80). These encouraging results have prompted the initiation of the Phase III STELLAR-303 trial, further exploring this therapeutic approach.

### 3.2 ICIs plus radiotherapy

Radiotherapy, like chemotherapy, can promote the release of tumor antigens and remodel the TME, enhancing systemic immunity through the abscopal effect (81, 82). Radiation-induced upregulation of cell surface molecules on tumor cells further augments the cytotoxic activity of NK cells and T cells (83). The release of type I interferons enhances T-cell priming and dendritic cell activation, contributing to a robust antitumor immune response (84, 85). These immunostimulatory effects make radiotherapy a valuable adjunct to ICIs in pMMR/MSS CRC.

A Phase II randomized trial reported impressive results, with short-course radiotherapy combined with the PD-1 inhibitor toripalimab and CAPOX achieving a complete response (CR) rate of 58.1% in patients with locally advanced rectal cancer (LARC) (86). Another study evaluated the efficacy and safety of tislelizumab (a PD-1 inhibitor) combined with chemoradiotherapy in LARC patients, demonstrating a CR rate of 40.0% (87). Although these results are promising, larger-scale trials are needed to validate these findings. Neoadjuvant chemoradiotherapy (NACRT) with or without sintilimab (a PD-1 inhibitor) achieved a CR rate of 44.8% in pMMR LARC patients, significantly higher than the 26.9% observed in the control group (88). Immunohistochemical analysis suggested that PD-L1-positive patients may derive greater benefit from this treatment, highlighting the importance of biomarker-driven patient selection.

A neoadjuvant therapy study involving 44 LARC patients treated with preoperative chemoradiotherapy (CRT) and nivolumab monotherapy reported 3-year relapse-free survival (RFS) outcomes. In MSS patients receiving CRT, those with high expression of PD-L1, PD-1, CTLA-4, and Ki-67, as well as an elevated CD8/eTreg ratio, exhibited a higher trend of 3-year RFS (89). These findings underscore the potential of combining radiotherapy with ICIs in pMMR/MSS CRC and suggest that neoadjuvant strategies may become a standard treatment option for this patient population.

### 3.3 ICIs plus anti-angiogenic agents

Anti-angiogenic agents, primarily tyrosine kinase inhibitors (TKIs) and antibodies targeting the vascular endothelial growth factor (VEGF)/VEGF receptor (VEGFR) pathway have demonstrated synergistic effects when combined with ICIs (90, 91). VEGF not only promotes angiogenesis but also exerts direct immunosuppressive effects by recruiting MDSCs and Tregs while inhibiting T-cell function (92, 93). Anti-angiogenic drugs, such as bevacizumab and ramucirumab, normalize tumor vasculature, improve blood perfusion, and alleviate hypoxia within the TME (94, 95). Vascular normalization enhances immune cell infiltration and reduces endothelial cell-induced T-cell apoptosis, creating a favorable environment for ICIs to exert their effects (96).

BD0801, a humanized anti-VEGF monoclonal antibody, has shown superior antitumor effects compared to bevacizumab in preclinical models, likely due to its potent VEGF/VEGFR blockade and inhibitory effects on human umbilical vein endothelial cells (97). The REGOMUNE trial, a Phase II study, evaluated the safety and efficacy of regorafenib combined with avelumab in MSS CRC patients, demonstrating good tolerability and no unexpected adverse events (98). Biomarker analysis revealed that patients with higher CD8^+^ T-cell infiltration experienced improved median OS and PFS, further supporting the combination of anti-angiogenic agents with ICIs (98). Fruquintinib, a highly selective TKI targeting VEGFR1, 2, and 3, has shown promising results in mCRC patients who have undergone at least two prior lines of chemotherapy. An international Phase III double-blind trial reported a median OS of 9.3 months and a median PFS of 3.7 months in the fruquintinib group, significantly higher than the 6.6 months and 1.8 months observed in the placebo group (99). These findings suggest that fruquintinib represents a valuable therapeutic option for mCRC patients. Other TKIs, including anlotinib (100–102) and sorafenib (103), have also shown potential in CRC treatment. Additionally, the emergence of bispecific antibodies, such as ivonescimab (anti-PD-1/VEGF-A), has expanded the therapeutic arsenal. A Phase Ia clinical trial demonstrated the promising antitumor activity of ivonescimab, with further studies underway to evaluate its efficacy in combination with other therapies (104).

However, prolonged VEGF/VEGFR blockade may lead to excessive vascular pruning, resulting in reduced drug distribution and hypoxia. Therefore, the therapeutic efficacy of anti-angiogenic agents depends on achieving a balance between vascular normalization and immune activation (96). Exploring triple therapy combinations involving anti-angiogenic agents, ICIs, and other modalities may further modulate the TME and enhance treatment outcomes.

### 3.4 ICIs plus MEK inhibitors

The mitogen-activated protein kinase (MAPK) pathway regulates critical cellular processes, including proliferation, differentiation, and apoptosis. Dysregulation of MAPK signaling contributes to uncontrolled cell growth and tumor development, making MEK a clinically relevant target (105, 106). MEKi can reduce the release of immunosuppressive factors and decrease the recruitment of immunosuppressive cells. Preclinical data suggest that MEK inhibition upregulates PD-L1 and MHC-I expression, facilitating the subsequent blockade of the PD-1/PD-L1 pathway (107–109).

A Phase Ib trial evaluated the combination of atezolizumab and cobimetinib (a MEK inhibitor) in patients with advanced solid tumors (110). Among the 84 enrolled mCRC patients (62 pMMR/MSS and 2 dMMR/MSI−H), only seven achieved confirmed responses. Diarrhea was the most frequent adverse event. The regimen demonstrated suboptimal safety, with grade 3–4 treatment−related adverse events occurring in 44% of patients and approximately 70% requiring treatment discontinuation or dose reduction due to intolerability (110). Although initial synergistic activity was observed in mCRC, it was not substantiated in a subsequent Phase III trial. Consequently, despite cobimetinib’s potential to modulate the tumor microenvironment, the atezolizumab–cobimetinib combination proved insufficient to overcome immunotherapy resistance in MSS mCRC patients.

Similarly, the Phase III IMblaze370 trial compared atezolizumab + cobimetinib and atezolizumab monotherapy versus regorafenib in the third−line setting for mCRC2. This study enrolled 363 patients (with dMMR/MSI−H recruitment capped at ≤ 5%) and used OS as the primary endpoint (111). IMblaze370 failed to meet its primary endpoint, demonstrating no significant difference in OS, ORR, or PFS for either atezolizumab−containing arm compared with regorafenib (111). These results indicate that combining MEK inhibitors with immune checkpoint inhibitors offers no substantial clinical benefit in pMMR/MSS CRC and warrants further investigation.

### 3.5 Other prospective combinations

#### 3.5.1 TGF-β: a double-edged sword in CRC

TGF-β is an pleiotropic cytokine whose signal is transduced from membrane to nucleus by SMAD proteins (112). Through the canonical TGF-β/SMAD4 axis, it governs virtually every facet of CRC-initiation, growth, apoptosis, differentiation and dissemination-yet its biological output is context-dependent (113). In early lesions, intact SMAD4 enforces cytostasis by inducing cell-cycle arrest and apoptosis, casting TGF-β as a bona-fide tumor suppressor (113, 114). Once genomic instability erodes this circuitry, malignant cells co-opt the pathway: autocrine TGF-β production fuels immune evasion, neovascularization and metastatic spread (113). Consequently, TGF-β overexpression marks a pivotal switch from tumor containment to progression (114, 115). Early clinical data now show that pharmacological blockade of TGF-β signaling dismantles this immunosuppressive scaffold, amplifies checkpoint-inhibitor efficacy and establishes a self-reinforcing loop of T-cell activation (112, 116). Dual targeting of TGF-β and immune checkpoints therefore constitutes a rational, mechanism-based strategy for microsatellite-stable CRC.

Bintrafusp alfa, a bifunctional fusion protein targeting PD-L1 and TGF-β, has shown promise in various cancers, including CRC (117), lung cancer (118), biliary tract cancer (119), and cervical cancer (120). Although a Phase III trial in advanced lung cancer was terminated early due to lack of superiority over pembrolizumab (121), preclinical studies suggest that bintrafusp alfa can reprogram the TME to overcome immune evasion and reduce radiation-induced fibrosis, highlighting its potential in combination therapies (122). A Phase I/Ib trial explored the use of NIS793, an anti-TGF-β monoclonal antibody, in combination with spartalizumab (a PD-1 inhibitor) in advanced solid tumors, demonstrating the potential for further development (123).

#### 3.5.2 Gut microbiota

Gut microbiota and their metabolites play a crucial role in modulating host immunity, particularly through bile acid metabolism and short-chain fatty acid production. Dysbiosis of the gut microbiota has been linked to CRC development and progression (124, 125). Emerging evidence suggests a strong association between gut microbiota composition and immunotherapy efficacy, including effects on treatment response and toxicity (126–128). In chemo-refractory non-small cell lung cancer and RAS wild‐type (WT) mCRC patients treated with cetuximab and avelumab, the presence of butyrate-producing bacterial strains, such as Agathobacter M104/1 and Blautia SR1/5, was associated with improved treatment outcomes (129).

Furthermore, gut microbiota is emerging as both modulators and biomarkers of immunotherapy efficacy. In advanced non-small-cell lung cancer receiving chemo-immunotherapy, patients treated with nivolumab plus ipilimumab and platinum doublet (NIC) exhibited higher baseline abundances of Faecalibacterium and Butyricicoccus compared with those receiving pembrolizumab plus platinum doublet (PC); this microbial signature was associated with superior overall survival (130). This analytical approach was similarly employed in a recent trial investigating atezolizumab plus bevacizumab (Atz/Bev) for recurrent mesothelioma (131). Collectively, these findings underscore the potential of gut flora to serve as predictive indicators of response in colorectal and other cancers treated with immune-based regimens. Even more exciting is that fecal microbiota transplantation (FMT) has shown promise in enhancing immunotherapy efficacy and overcoming resistance to ICIs in melanoma patients (132, 133). These findings underscore the potential of modulating the gut microbiota to improve immunotherapy outcomes in CRC.

Combination therapies involving ICIs for pMMR/MSS CRC face significant challenges. The tumor microenvironment often resists immune activation, rendering ICIs less effective. Moreover, potential synergistic effects and irAEs require careful management. Additionally, many primary and secondary resistance mechanisms are present in the tumor microenvironment that prevent the efficacy of ICIs. For example, impairments in antigen presentation machinery and IFN-γ signaling pathways can lead to resistance to immune checkpoint blockade therapy. The integration of ICIs with other treatment approaches holds promise in enhancing the efficacy of immunotherapy in CRC patients, but further research is needed to confirm the synergistic effects of these combinations.

## 4 Cancer vaccines

Cancer vaccines hold promise for both pMMR/MSS CRC and dMMR/MSI CRC. Unlike dMMR/MSI CRC, pMMR/MSS CRC has a lower mutation load but still harbors specific mutations. Recent advances in vaccine development are reshaping therapeutic paradigms for CRC, with neoantigen-targeted vaccines emerging as particularly transformative agents. Conventional vaccines targeting Tumor-Associated Antigens (TAAs) face inherent limitations due to shared expression in normal tissues, raising risks of autoimmune sequelae (134). In contrast, neoantigen vaccines leverage tumor-specific somatic mutations to elicit precise antitumor immunity while sparing healthy tissues, thereby overcoming central tolerance mechanisms and enabling personalized therapeutic strategies (135, 136). The efficacy of such approaches correlates strongly with TMB, where hypermutated tumors exhibit enhanced immunogenic potential (137). While vaccines enrich the presentation of tumor-specific antigens, immune checkpoint inhibitors (ICIs) relieve immune system suppression. Their synergistic effects enhance anti - tumor immunity. Research is now moving toward combining cancer vaccines with ICIs rather than using vaccines alone. For instance, in the NCT04041310 trial conducted on dMMR/MSI CRC patients, the combination of cancer vaccines and ICIs was explored. Furthermore, advancements in delivery platforms spanning nucleic acid-based vectors (DNA/RNA), antigen-presenting cell (APC) systems, and engineered bacterial carriers have substantially expanded the therapeutic arsenal for CRC management (138). (**Table 6**).

**Table 6:** Clinical trials based on other immunotherapy for the treatment of colorectal cancer.

Type of immunotherapy	ClinicalTrials.Gov Identifier	Combinatorial agent(s)	Phase	Primary endpoint	Status
Oncolytic Virus Therapy	NCT05492682		Phase 1	Tolerability,Safety	Recruiting
	NCT03740256		Phase 1	DLTs	Recruiting
	NCT05860374		Phase 1	TEAEs	Recruiting
	NCT04046445	Ezabenlimab;ATP128	Phase 1	TEAEs,PFS	Active
	NCT03206073	Durvalumab;Tremelimumab	Phase 1,2	TEAEs	Completed
	NCT03225989		Phase 1,2	Safety	Completed
	NCT01394939	Irinotecan	Phase 1,2	Radiographic Response Rate	Completed
	NCT05733611	Bevacizumab;Atezolizumab	Phase 2	ORR	Active
Adoptive Cell Therapy	NCT05902520	IL-2	Phase 1	Safety	Recruiting
	NCT06718738		Phase 1	TEAEs	Recruiting
	NCT05089266		Phase 1	DLTs	Recruiting
	NCT05240950		Phase 1	Safety,Effectiveness	Recruiting
	NCT05396300		Phase 1	Safety,Tolerability	Active
	NCT03431311		Phase 1,2	TEAEs	Terminated
	NCT05736731		Phase 1,2	DLTs,RP2D,ORR	Recruiting
	NCT05759728		Phase 1,2	Safety,Best response	Recruiting
	NCT02487992	S-1,Bevacizumab	Phase 2	OS	Active
	NCT02419677	Radiofrequency ablation	Phase 2,3	RFS	Completed
Matrix-depletion Therapy	NCT02546531	Defactinib,Pembrolizumab	Phase 1	RP2D,MTD	Completed
	NCT03875820	VS-6766(a Dual RAF/MEK Inhibitor)	Phase 1	RP2D,TEAEs	Active
	NCT04439331		Phase 2	ORR	Active
	NCT06369259	Defactinib;Cetuximab,Avutometinib	Phase 2	Safety,TEAEs	Recruiting

Preclinical validation in CRC murine models demonstrated that engineered neoantigen vaccines induce robust antitumor activity, significantly suppressing metastatic progression and extending overall survival through tumor-specific T-cell priming (139). Translational studies in patients with recurrent or metastatic MSS CRC revealed clinically meaningful stratification: responders exhibiting neoantigen-specific immunity achieved superior progression-free survival (PFS:19 vs 11 months) compared to non-responders (140). Parallel breakthroughs in renal cell carcinoma, a malignancy sharing immunological features with MSS CRC, demonstrated universal neoantigen-specific T-cell activation across nine treated patients, with no disease recurrence or dose-limiting toxicities observed during follow-up (141). These findings have galvanized clinical exploration, with ongoing trials such as NCT05141721 evaluating neoantigen vaccines GRT-C901/GRT-R902 in combination with ICIs, with PFS as the primary endpoint in phase III evaluation.

Despite the high specificity and immunogenicity potential of novel antigen vaccines for pMMR/MSS CRC and dMMR/MSI CRC, significant barriers are still present (140, 142). The customization required for these vaccines demands intensive individualized analysis, which is not only cost-prohibitive but also time-intensive, thereby potentially deferring critical treatment initiation. Regarding clinical trials, while early-stage studies have yielded encouraging results, these outcomes have not been consistently replicated in large-scale Phase III trials. This discrepancy may stem from a variety of factors, including trial design limitations, suboptimal patient cohort selection, and the application of efficacy evaluation criteria that may not fully capture the complex biological and clinical impacts of new antigen vaccines.

## 5 Other immune-related therapies

In the realm of CRC immunotherapy, emerging breakthroughs such as oncolytic virotherapy, ACT, and matrix-depletion therapy have demonstrated significant therapeutic potential (**Table 7**). Although their mechanisms of action differ, these strategies collectively enhance immune function to combat CRC, each with distinct advantages and limitations. Oncolytic viruses (OVs) exhibit a remarkable ability to augment T-cell-mediated cytotoxicity, yet they face challenges such as inefficient delivery and limited tumor penetration (143, 144). ACT offers durable anti-tumor effects but is hindered by high costs and the risk of T-cell exhaustion (145, 146). Matrix-depletion therapy, on the other hand, disrupts the dense extracellular matrix (ECM) surrounding tumors, improving drug penetration and enhancing the efficacy of other treatments (147). The following sections will elaborate on these three immunotherapeutic approaches.

**Table 7:** Clinical trials based on neoantigen vaccines for the treatment of colorectal cancer.

ClinicalTrials.Gov Identifier	Combinatorial agent(s)	Phase	Primary endpoint	Status
NCT03948763	Pembrolizumab	Phase 1	DLTs	Terminated
NCT06195384		Phase 1	Safety	Recruiting
NCT06496373		Phase 1	Reaction of antigen-specific T cells	Recruiting
NCT05359354		Not Applicable	MTD,DLTs	Recruiting
NCT05916248	Pembrolizumab	Phase 1	MTD,DLTs,ORR,DCR	Recruiting
NCT05940181	Sintilimab	Interventional	MTD,DLTs,Safety	Recruiting
NCT06497010		Phase 1	RP2D	Recruiting
NCT03639714	Nivolumab;Ipilimumab	Phase 1,2	TEAEs,DLTs,ORR, RP2D	Completed
NCT06751966		Not Applicable	ORR,DCR,Safety	Recruiting
NCT06751914		Not Applicable	ORR,DCR,Safety	Recruiting
NCT04147078		Phase 1	DFS	Recruiting
NCT04117087	Nivolumab;Ipilimumab	Phase 1	Safety,Fold change in CD8 and CD4 T cells	Recruiting
NCT02600949		Phase 1	Feasibility,TEAEs	Recruiting
NCT03552718		Phase 1	Safety,RP2D	Active
NCT04041310	Pembrolizumab	Phase 1,2	DLTs,ORR,Safety, Tolerability	Active
NCT03953235	Nivolumab;Ipilimumab	Phase 1,2	TEAEs,DLTs, ORR, RP2D	Completed
NCT04912765	Nivolumab	Phase 2	PFS	Recruiting
NCT06522919	Pembrolizumab	Phase 2	ORR,PFS	Recruiting
NCT05141721	Atezolizumab;Fluoropyrimidine;Bevacizumab;Ipilimumab	Phase 2,3	ctDNA,PFS	Active

### 5.1 Oncolytic virotherapy

OVs exert their antitumor effects through two distinct yet complementary mechanisms (1): direct cytopathic destruction of malignant cells via selective intracellular replication and subsequent cell lysis, and (2) indirect immunostimulatory effects mediated by the massive release of TAAs and neoantigens following viral-induced oncolysis, which initiates systemic antitumor immune responses (12). Importantly, the presentation of viral antigens additionally triggers robust antiviral immune responses that exhibit cross-reactivity with tumor cells, thereby establishing a bimodal attack on neoplastic tissues (148).

A groundbreaking clinical advance was achieved by Zhao Yongxiang’s team through the development of NDV-GT, a recombinant Newcastle disease virus engineered to express porcine α1,3-galactosyltransferase (α1,3GT). This modification induces tumor-specific hyperacute immune rejection via ectopic α-gal epitope expression (149). In a phase I/II trial involving 23 patients with advanced malignancies, NDV-GT elicited a DCR of 90.00% (18/20) with no severe adverse events. Notably, three CRC patients discontinued treatment due to COVID-19 pandemic-related logistical constraints, underscoring real-world challenges in clinical trial execution (149).

Genetic modification of adenoviral vectors enables precise modulation of the TME (150). Rongye Jing and colleagues developed rAd.mDCN.mCD40L, a novel oncolytic adenovirus co-expressing CD40 ligand and decorin, a stromal remodeling agent. In CT26 CRC models, this construct demonstrated potent suppression of hepatic metastasis (151). Another studies revealed that adenoviruses encoding glypican-3 core proteins synergized with NK cell adoptive transfer, enhancing NK-mediated tumor cytotoxicity and intratumoral lymphocyte infiltration in CRC xenografts (152). These findings validate OVs as potent adjuvants for cell-based immunotherapies through multidimensional immune activation (152–154).

While FOLFOXIRI chemotherapy remains a cornerstone in mCRC management, its limited efficacy has prompted exploration of OV-chemotherapy combinations. Girod et al. demonstrated enhanced tumoricidal activity in Colo320 CRC cells through co-administration of coxsackievirus B3 PD-H and chemotherapeutic agents, with combination therapy surpassing monotherapy efficacy via virus-mediated chemosensitization (155). To address targeting limitations and therapeutic resistance, a novel approach utilizing mesenchymal stem cell (MSC)-delivered coxsackievirus A21 achieved robust antitumor effects in CRC murine models, highlighting the potential of cellular vehicles for precise viral delivery (156).

Furthermore, the release of TAAs and cytokines following oncolysis is considered a potential mechanism for enhancing ICIs (157). Several clinical studies (158, 159) have evaluated the combination of oncolytic virotherapy with ICIs in CRC, demonstrating favorable clinical activity and manageable toxicity. In a first-in-human signal-seeking study, 34 patients with proficient-mismatch-repair/microsatellite-stable pMMR/MSS CRC received the oncolytic vaccinia virus pexastimogene devacirepvec (Pexa-Vec) in tandem with the PD-L1 blocker durvalumab, with or without the CTLA-4 antagonist tremelimumab (158). Although the combination was well tolerated, its antitumor impact was marginal: median progression-free survival edged from 2.1 to 2.3 months—incremental gains that fall squarely within the efficacy corridor of existing FDA-approved third-line therapies for mCRC (158). Separately, intratumoral delivery of the engineered herpes simplex virus-2 derivative OH2, combined with the PD-L1 inhibitor LP002, yielded an exceptional responder: a single responder experienced a 313-day remission and remains alive at 499 days (159). Such a solitary signal, however, cannot escape the statistical noise inherent to a four-patient cohort; confirmation awaits adequately powered, prospectively designed trials (159).

But, oncolytic virotherapy for CRC faces three main challenges. First, delivery difficulties. Most CRC tumors are internal, making direct injection tough. Second, immune response balance. Pre-existing immunity can block oncolytic virus efficacy. Repeated injections may trigger neutralizing antibodies. Third, lack of predictive biomarkers. Identifying patients most likely to benefit from oncolytic virotherapy is challenging due to the absence of reliable biomarkers. These issues limit oncolytic virotherapy’s effectiveness and broader application in CRC treatment (160).

### 5.2 ACT

ACT represents a highly innovative approach to cancer treatment, involving the extraction, modification, and expansion of immune cells *ex vivo*, followed by re-infusion into patients to enhance anti-tumor immunity. Among various ACT modalities, NK cell therapy, which activates innate immunity, and chimeric antigen receptor T (CAR-T) cell therapy, which offers precise tumor targeting, are the most prominent (161). In CRC, cytokine-induced killer (CIK) cell therapy and CAR-T cell therapy have shown promising results, while newer approaches, such as T-cell receptor-engineered T cells and CAR-macrophages, hold significant potential (161). As technology advances and more clinical trials are conducted, ACT is expected to further improve CRC treatment outcomes, offering hope for better patient survival.

CIK cells are a heterogeneous population of immune effector cells generated by stimulating and expanding peripheral blood mononuclear cells with cytokines ex vivo (162). This population includes conventional T cells, NK cells, and NKT-like cells, with NKT-like cells being the primary effectors that can recognize and kill tumors in an MHC-unrestricted manner (162). A meta-analysis of 70 clinical trials involving 6,743 CRC patients demonstrated that CIK cell therapy significantly improved clinical outcomes, particularly in terms of quality of life and survival (163). Most studies (66 trials) combined CIK therapy with chemotherapy (e.g., FOLFOX or XELOX regimens) to enhance chemotherapy efficacy and mitigate its side effects. Among these, 45 trials administered CIK cells concurrently with chemotherapy, with infusion timing varying across studies: early (3 trials), mid-chemotherapy (7 trials), late (13 trials), and unspecified (22 trials). Future studies should explore optimal infusion schedules to maximize therapeutic benefits. Additionally, personalized CIK cell therapies tailored to patients’ genetic mutations and immune profiles are under development.

CAR-T cell therapy involves genetically modifying patients’ T cells to express a chimeric antigen receptor (CAR) targeting specific tumor antigens. Upon re-infusion, CAR-T cells recognize and bind to tumor surface antigens, releasing cytokines that induce tumor cell apoptosis (164). In CRC, current trials focus on identifying suitable tumor antigens and improving CAR-T cell efficacy and safety. Common targets include carcinoembryonic antigen (CEA) and NKG2D ligands. Several trials (NCT02349724, NCT02416466, NCT02850536) have assessed the safety of CAR-T cells in CEA-positive patients. Notably, NCT02349724 showed that one patient survived for 23 months with elevated serum IFN-γ levels and no grade 3 or higher adverse events (165). Similarly, no severe adverse events were observed in the other trials, confirming the safety of CAR-T infusion (166, 167). Furthermore, NCT02850536 demonstrated improved CAR-T cell delivery to the liver, resulting in increased OS (167). Despite these promising advances, CAR-T therapy in CRC remains limited and requires further exploration to enhance its efficacy.

ACT for CRC faces several key challenges. The immunosuppressive TME of solid tumors poses a significant barrier to ACT efficacy by inhibiting T cell function (168). Additionally, identifying suitable targets that balance safety and efficacy is challenging. ACT is also associated with substantial toxicities, such as cytokine release syndrome, neurotoxicity, and target-related adverse effects (169). The generation of tumor-specific lymphocytes for each patient is technically demanding and economically costly, which limits the widespread application of ACT. Furthermore, T cell exhaustion, characterized by the loss of effector function and self-renewal capacity, further diminishes treatment effectiveness (169). These complexities collectively restrict the clinical application of ACT in CRC.

### 5.3 Matrix-depletion therapy

Matrix-depletion therapy is an emerging strategy that targets the ECM within the TME. This approach primarily addresses collagen metabolism dysregulation, aberrant activation of matrix metalloproteinases, and the dysregulated expression of factors such as TGF-β (170–172). In CRC, these imbalances are closely linked to TME reprogramming, making ECM components potential therapeutic targets (173, 174). Although matrix-depletion therapy is still in its early stages in CRC, it has shown promise in other stroma-rich malignancies, such as pancreatic cancer.

One key ECM component, hyaluronic acid, plays a critical role in tumor progression. A Phase II trial in metastatic pancreatic cancer patients tested the combination of pegvorhyaluronidase alfa (PEGPH20) with nab-paclitaxel, gemcitabine plus nab-paclitaxel, and gemcitabine alone (175). The results demonstrated that PEGPH20 significantly prolonged PFS and increased ORR, particularly in patients with high hyaluronic acid expression (175). However, a subsequent Phase III trial failed to show benefits in OS or PFS with PEGPH20, leading to its discontinuation in development (176).

Another promising target in matrix-depletion therapy is the Hedgehog signaling pathway, which is implicated in stromal formation. Inhibition of this pathway has been shown to reduce stromal density (177). A Phase II trial combining a Hedgehog pathway inhibitor with cemiplimab in metastatic basal cell carcinoma showed promising anti-tumor activity and safety (178). However, results in sarcoma and myeloma were less encouraging, necessitating further exploration in CRC (179, 180).

Focal adhesion kinase (FAK), a tyrosine kinase involved in integrin signaling, has emerged as another therapeutic target (181). A Phase II trial evaluating a FAK inhibitor in meningioma reported a 33% six-month PFS rate, highlighting its potential as a therapeutic strategy (182).

In summary, while matrix-depletion therapy shows promise, it faces several challenges, including limited specificity and tumor heterogeneity. Moreover, ECM degradation may facilitate circulating tumor cell dissemination, underscoring the need for further safety and efficacy evaluations in CRC.

## 6 Conclusion and future perspectives

There is no doubt that immunotherapy has achieved remarkable success in CRC treatment. ICIs have made significant strides, while cancer vaccines, oncolytic virotherapy, ACT, and matrix-depletion therapy hold immense potential. Each modality offers unique advantages but also presents challenges. For instance, prolonged ICI use may lead to resistance, and cancer vaccines face hurdles such as insufficient immunogenicity and tumor heterogeneity. OVs exhibit high oncolytic specificity but are limited by the availability of viral vectors and require further evaluation of their efficacy and safety. ACT, though promising, remains unpredictable in its outcomes. While certain matrix-modulating agents can improve the CRC immune microenvironment, the complex interactions among ECM components complicate therapeutic strategies.

In most studies, ICI monotherapy or ICI-ICI combination therapy had an ORR of less than 10% in patients with MSS/pMMR CRC. Thus, addressing the majority of patients with MSS/pMMR CRC is a key challenge (183). To enhance treatment efficacy, various ICI-based strategies, including combinations with radiotherapy, chemotherapy, anti-angiogenic agents, and MEK inhibitors, have been tested in this subgroup. Additionally, targeting TGF-β in combination with ICIs and leveraging the immunomodulatory role of gut microbiota are promising avenues. Breakthroughs in neoantigen vaccines and the emergence of next-generation immune checkpoints such as LAG-3, TIM-3, and TIGIT have further invigorated the field (141, 184, 185).

In conclusion, there are still some challenges that need to be overcome (1): Investigating the crosstalk among various immunosuppressive cells in the CRC immune microenvironment (2); Identifying precise predictive biomarkers (3); Optimizing combination therapy strategies in terms of efficacy, safety, and personalization. By deepening our understanding of the biology and immune microenvironment of CRC, and by integrating new technologies and treatments, we can advance CRC treatment and improve patient outcomes.
