# Correction: Emerging strategies in colorectal cancer immunotherapy: enhancing efficacy and survival

**DOI:** 10.3389/fimmu.2025.1727580

**Published:** 2025-11-07

**Authors:** Yu Zhang, Haixia Guan, Xixuan Feng, Mengyan Liu, Jinhuan Shao, Mengchi Liu, Jialei He, Yahui Jin, Jinglin Zhu, Chunli Zheng

**Affiliations:** Yan’an Medical College, Yan’an University, Yan’an, Shaanxi, China

**Keywords:** colorectal cancer immunotherapy, immune checkpoint inhibitors, cancer vaccines, adoptive cell therapy, matrix-depletion therapy

There was a mistake in **Table 7** as published. The phases corresponding to NCT06751966 and NCT06751914 were omitted. The corrected **Table 7** appears below.

**Table 7:** Clinical trials based on neoantigen vaccines for the treatment of colorectal cancer.

ClinicalTrials.Gov identifier	Combinatorial agent(s)	Phase	Primary endpoint	Status
NCT03948763	Pembrolizumab	Phase 1	DLTs	Terminated
NCT06195384		Phase 1	Safety	Recruiting
NCT06496373		Phase 1	Reaction of antigen-specific T cells	Recruiting
NCT05359354		Not Applicable	MTD,DLTs	Recruiting
NCT05916248	Pembrolizumab	Phase 1	MTD,DLTs,ORR,DCR	Recruiting
NCT05940181	Sintilimab	Interventional	MTD,DLTs,Safety	Recruiting
NCT06497010		Phase 1	RP2D	Recruiting
NCT03639714	Nivolumab;Ipilimumab	Phase 1,2	TEAEs,DLTs,ORR, RP2D	Completed
NCT06751966		Not Applicable	ORR,DCR,Safety	Recruiting
NCT06751914		Not Applicable	ORR,DCR,Safety	Recruiting
NCT04147078		Phase 1	DFS	Recruiting
NCT04117087	Nivolumab;Ipilimumab	Phase 1	Safety,Fold change in CD8 and CD4 T cells	Recruiting
NCT02600949		Phase 1	Feasibility,TEAEs	Recruiting
NCT03552718		Phase 1	Safety,RP2D	Active
NCT04041310	Pembrolizumab	Phase 1,2	DLTs,ORR,Safety, Tolerability	Active
NCT03953235	Nivolumab;Ipilimumab	Phase 1,2	TEAEs,DLTs, ORR, RP2D	Completed
NCT04912765	Nivolumab	Phase 2	PFS	Recruiting
NCT06522919	Pembrolizumab	Phase 2	ORR,PFS	Recruiting
NCT05141721	Atezolizumab;Fluoropyrimidine;Bevacizumab;Ipilimumab	Phase 2,3	ctDNA,PFS	Active

The original version of this article has been updated.

